# Supplementary material for: Fourteen-Day Gemcitabine-Docetaxel Chemotherapy Is Effective and Safer Compared to 21-Day Regimen in Patients with Advanced Soft Tissue and Bone Sarcoma
Source: Cancers (Basel). 2021 Apr 20;13(8):1983. doi: 10.3390/cancers13081983 (PMC8074251; doi:10.3390/cancers13081983)
Supplement: Supplementary file 1 [file cancers-13-01983-s001.zip › cancers-1125169-supplementary.pdf]

Supplementary Files

Fourteen-Day Gemcitabine-Docetaxel Chemotherapy Is Effective and Safer Compared to 21-Day Regimen in Patients with Advanced Soft Tissue and Bone Sarcoma

Minggui Pan, Maily K. Trieu, Manpreet Sidhu, Jeanette Yu, Tiffany Seto and Kristen Ganjoo

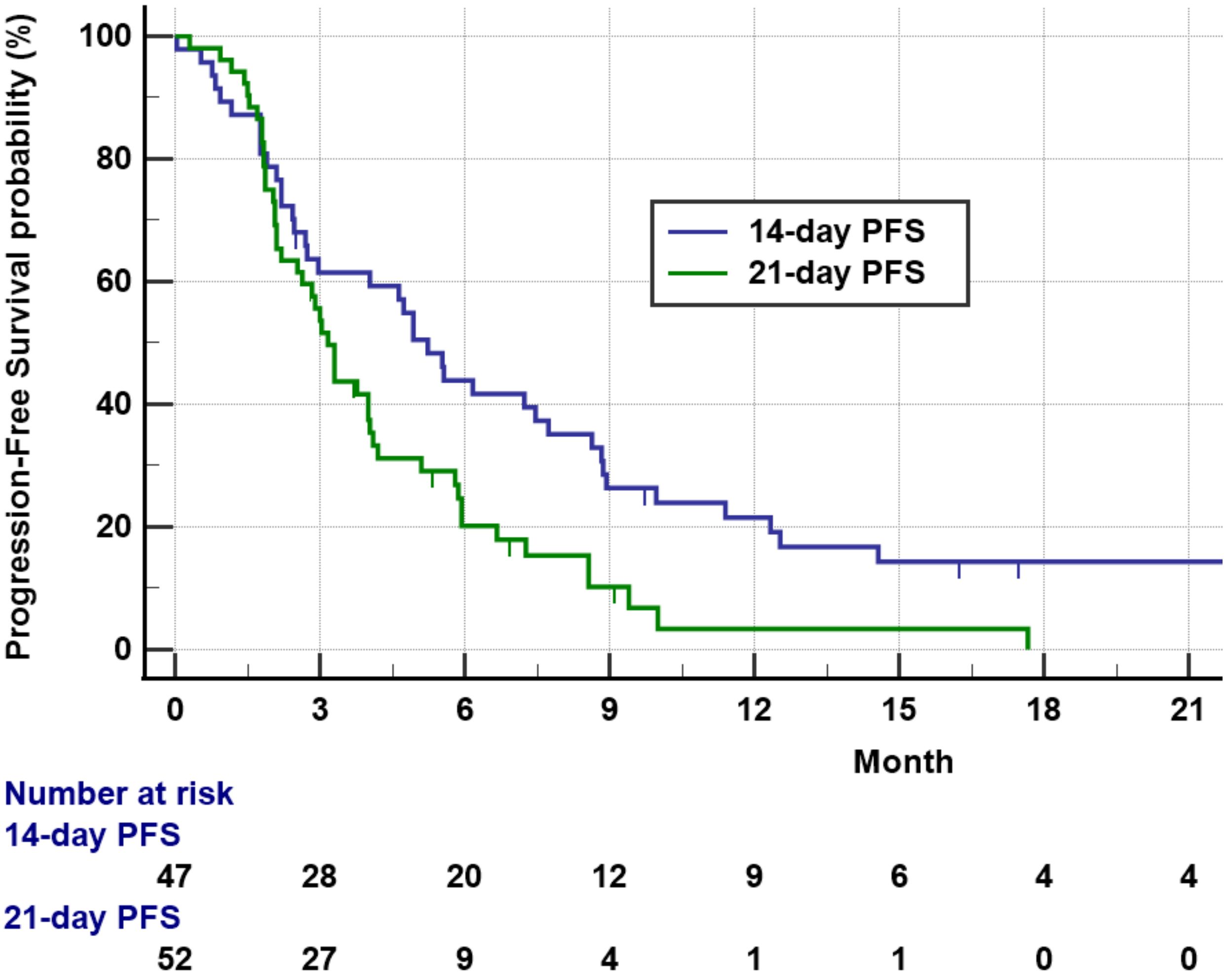

Figure S1. Kaplan-Meyer plot of PFS of patients treated with first-line 14-day or 21-day G-D regimen. Note the median PFS was 5.2 months versus 3.2 months for 14-day (blue) and 21-day (green) G-D regimen respectively ($p = 0.01$). Patients who had not progressed are marked as censored. Number of patients at risk is indicated in the bottom.

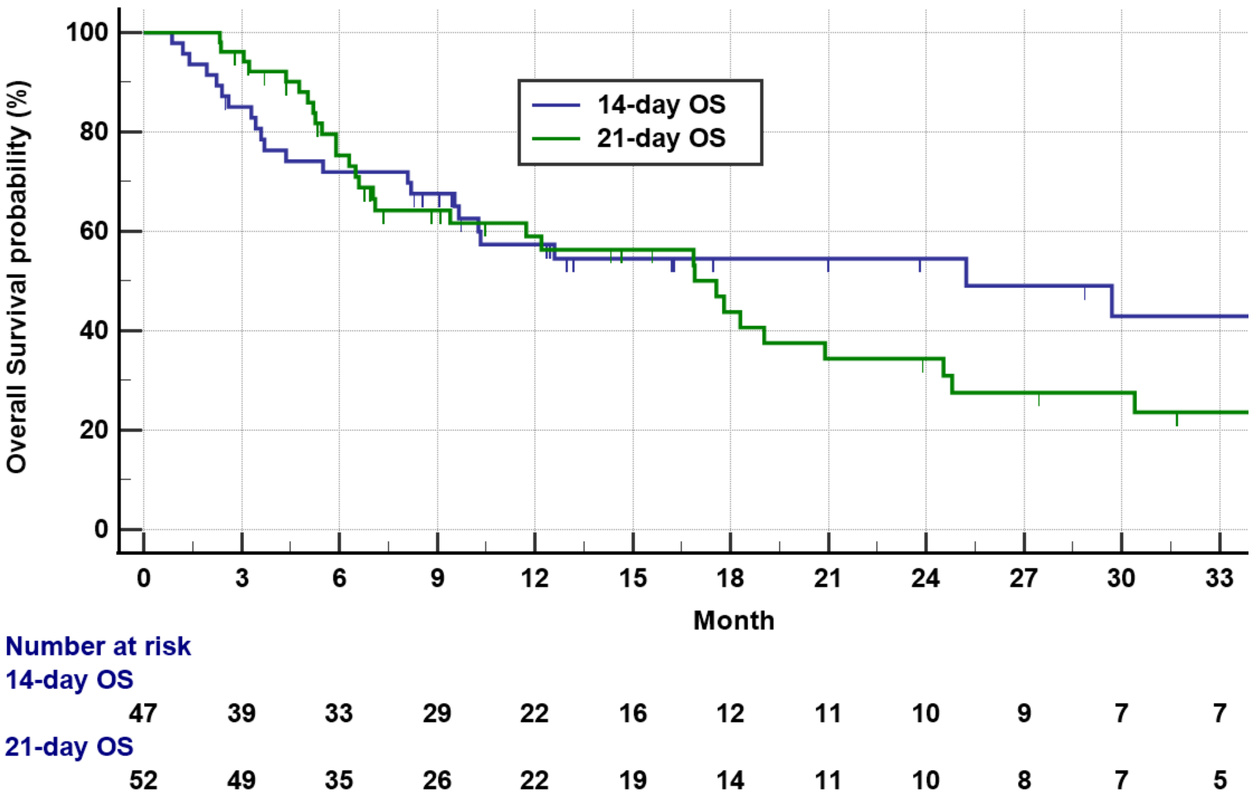

Figure S2. Kaplan-Meyer plot of OS of patients treated with first-line 14-day or 21-day G-D regimen. Note the median OS was 25.2 months versus 17.6 months for 14-day(blue) and 21-day (green) G-D regimen respectively ($p = 0.57$). Patients who were still alive are marked as censored. Number of patients at risk is indicated in the bottom.

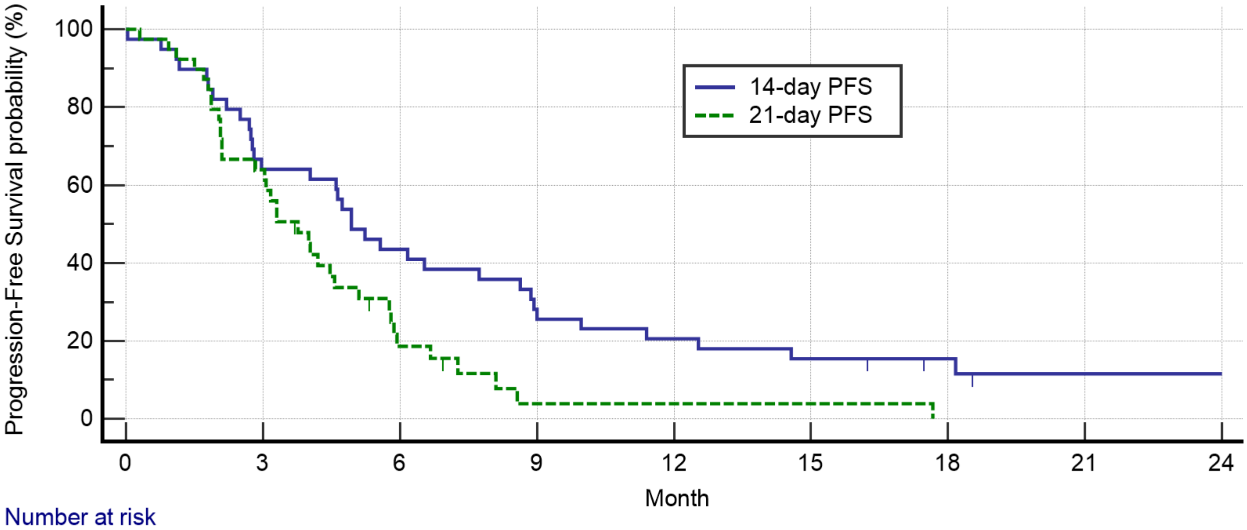

Number at risk

	0	3	6	9	12	15	18	21	24
14-day PFS	39	25	17	10	8	6	4	2	2
21-day PFS	39	24	6	1	1	1	0	0	0

Figure S3. Kaplan-Meyer plot of PFS of patients with advanced leiomyosarcoma (LMS) treated with 14-day (blue) versus 21-day (green) G-D regimen. Note $p = 0.01$ indicating better PFS for patients treated with 14-day G-D regimen compared to 21-day regimen. Patients who have not progressed are marked as censored. Number of patients at risk is indicated in the bottom.

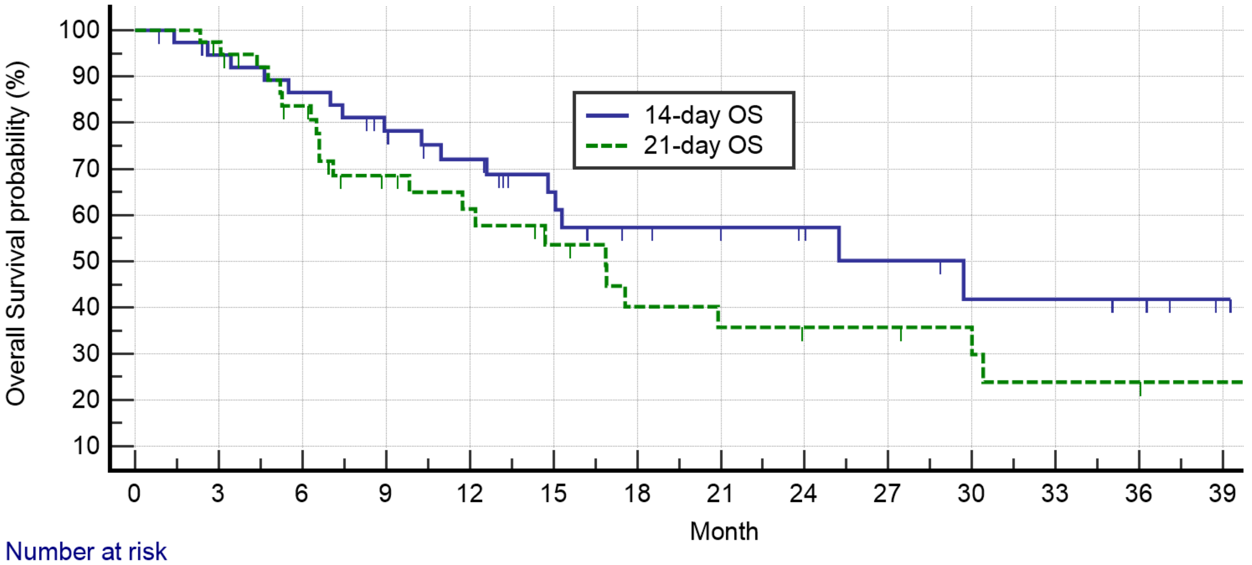

Number at risk

14-day OS	39	35	32	27	23	17	12	10	9	7	5	5	4	1
21-day OS	39	37	29	20	17	13	9	8	7	7	5	4	4	3

Figure S4. Kaplan-Meyer plot of OS of patients with advanced LMS treated with 14-day (blue) or 21-day (green) G-D regimen. Note $p = 0.2$ indicating no significant statistical difference between patients treated with either 14-day (blue) or 21-day (green) G-D regimen. Patients who were still alive are marked as censored. Number of patients at risk is indicated in the bottom.
